# Prevalence and impact of disability in women who had recently given birth in the UK

**DOI:** 10.1186/1471-2393-12-31

**Published:** 2012-04-27

**Authors:** Dana Šumilo, Jennifer J Kurinczuk, Maggie E Redshaw, Ron Gray

**Affiliations:** 1 Policy Research Unit in Maternal Health & Care, National Perinatal Epidemiology Unit, Department of Public Health, University of Oxford, Old Road Campus, Oxford, OX3 7LF, UK

**Keywords:** Disability, Limiting longstanding illness, Pregnancy, Millennium Cohort Study

## Abstract

**Background:**

Maternity services should take into account the needs of all women, including those related to disability. No reliable information, however, exists on the extent and characteristics of disability in this population in the UK. This brief report provides an overview of the prevalence of disability in women giving birth in the UK as measured by the presence of a limiting longstanding illness (LLI). The demographic, socio-economic, lifestyle and pregnancy related characteristics and child health outcomes are summarised to inform maternity and postnatal care service planning, and policy development.

**Methods:**

Secondary analysis of data on 18,231 mother-child pairs from the nationally representative UK Millennium Cohort Study. The baseline interviews with families were carried out in 2001-2002. The LLI prevalence in women who had recently delivered was estimated, and relevant characteristics and differences in outcomes compared using descriptive statistics taking into account the study design and non-response.

**Results:**

9.4% (95% CI 8.7-10.0) of women who had recently given birth reported having an LLI. Musculoskeletal, respiratory and mental disorders accounted for most of the health problems. A significantly higher proportion of women with an LLI received means-tested financial benefits, had no educational qualifications and suffered from intimate partner violence compared to women who did not have an LLI (49.3% vs 35.3%, 20.4% vs 15.0%, 6.0% vs 3.3%, respectively). They were also more likely to smoke throughout pregnancy than women without an LLI (29.2% vs 20.8%), have a preterm birth (10.9% vs 6.8%) and be lone parents (19.5% vs 13.9%). Only 25.6% of children of mothers with an LLI were breastfed for more than three months compared to 33.4% of infants of mothers who did not have an LLI. At the age of seven years, 12.0% of children of mothers with an LLI had an activity limiting health problem themselves compared to 6.2% of children of mothers without an LLI.

**Conclusions:**

Disability in women who had recently delivered is relatively common. It is associated with social and economic inequalities and worse pregnancy and child related outcomes. Apart from condition-specific support during and after pregnancy, disabled women may require extra help from health professionals to quit smoking, continue breastfeeding, and reduce intimate partner violence.

## Background

Maternity services should be accessible to all women and be designed to take into account their individual needs, including those related to disability [1]. Service planning for women with a disability requires information; there are, however, no reliable statistics on the proportion of women accessing antenatal care who are disabled.

The World Health Organisation, in the International Classification of Functioning, Disability and Health, uses the term ‘disability’ as an “umbrella term for impairments, activity limitations and participation restrictions” [2].There are different approaches to quantifying disability and its domains due to the complexity of the concept. The approach taken also depends on the purpose for which data are sought. The global estimate of disability prevalence in adults is between 16% and 19%, with a lower prevalence in younger age groups (9% in 18-49 year olds) [3].

This paper provides an overview of the prevalence of limiting longstanding illness, disability and infirmity in women giving birth in the UK. This way of quantifying limiting longstanding illness and disability in the general population has been widely used, for example with a similar question included in the national UK Census 2011 [4]. Even though it does not capture fully the multidimensionality of the concept of disability, it can provide useful baseline information on the extent of disability in women accessing maternity services for future maternity service planning and health policy development, leading to increased awareness about this population group, assessment of their needs, improved access to services and a reduction in the avoidable inequalities experienced by these women. This paper also provides a summary of the characteristics and differences in pregnancy and child health outcomes for women with and without a limiting longstanding illness.

## Methods

The MCS is a nationally representative prospective UK cohort study of children born during 2000-2002. The study is housed at the Centre for Longitudinal Studies (CLS), based at the Institute of Education, University of London and funded by the Economic and Social Research Council (ESRC), the consortium of Government Departments and the Wellcome Trust. Ethics approval for the MCS was granted from the Multi-Centre Research Ethics Committee in the UK. Secondary analysis of the publicly available Millennium Cohort Study (MCS) data was undertaken and therefore specific ethics approval for this work was not required. Full details of study design and data collection have been published previously.[5-7] The study sample was clustered geographically at the electoral ward level and disproportionately stratified to allow for adequate representation of families living in areas of high child poverty, ethnic minority populations and three smaller countries in the UK. The families were identified from the UK Child Benefit system which covers all UK-resident children except those whose residency status is uncertain or temporary (such as children of asylum seekers and members of foreign armed forces). In total, baseline information on 18,552 families and 18,818 infants was collected by trained interviewers during home visits when children were aged nine months, with further interviews at three, five and seven years.

Families where the natural mother was not the main respondent (n = 57) and families with twins and triplets (n = 256) were excluded from this analysis. When children were nine months old, mothers were asked:“Do you have a longstanding illness, disability or infirmity. By longstanding I mean anything that has troubled you over a period of time or that is likely to affect you over a period of time?” If the respondent answered “Yes” to this question they were asked “What is the matter with you?” and “Does this illness or disability limit your activities in any ways?” The limiting longstanding illnesses (LLIs) named by respondents were coded at the CLS using the tenth revision of the International Classification of Diseases (ICD-10). Eight responses were missing; therefore the total number of mother infant pairs included in this analysis was 18,231.

Data were analysed in Stata SE 11.1 (Stata Corporation, Texas, USA) using survey commands with sampling and non-response weights to adjust for the clustered sample design, unequal probability of being sampled and attrition between study years. Where the characteristics of the LLIs are summarised, this paper focuses on the first coded illness. Weighted proportions, their 95% confidence intervals (95% CIs) and p values of the Pearson χ^2^ statistic corrected for the survey design are reported.

## Results

The prevalence of any LLI among women who had given birth in 2000-2002 was 9.4% (95% CI 8.7-10.0). A total of 77.9% (95% CI 75.7-80.0) of mothers with an LLI reported having one such condition; 17.8% (95% CI 15.8-20.0) reported two LLIs; and 4.3% (95% CI 3.3-5.5) reported three or more. By far the most common group of LLIs in women who had recently given birth were diseases of the musculoskeletal system and connective tissue (Table 1), with back disorders being the most prevalent. Diseases of respiratory system (particularly asthma) and mental health problems (particularly recurrent depressive disorder) were also common. Blindness, visual impairment and hearing loss accounted for 1.0% of LLIs.

**Table 1:** The most common groups of limiting longstanding illnesses in women who had recently given birth

**Limiting Longstanding Illnesses**	% of total* (n = 1,705)
**Diseases of the musculoskeletal system and connective tissue,** including:	**29.3**
Back disorders	20.0
Arthritis	3.5
Other joint disorders	3.3
**Diseases of the respiratory system,** including:	**14.7**
Asthma	13.1
**Mental and behavioural disorders,** including:	**13.8**
Recurrent depressive disorder	9.3
**Diseases of the nervous system,** including:	**7.8**
Migraine	2.4
Epilepsy	1.8
**Diseases of the digestive system,** including:	**4.7**
Irritable bowel syndrome	1.2
**Endocrine, nutritional and metabolic diseases,** including:	**4.6**
Thyroid disorders	2.5
Diabetes mellitus	1.3
**Diseases of the circulatory system,** including:	**3.6**
Primary hypertension	1.2
**Diseases of the genitourinary system,** including:	**3.3**
Endometriosis	1.1

*Estimates are weighted for the survey design and non-response. % are calculated from the total of the first coded illnesses. Major ICD-10 groups contributing more than 3% of the total are summarised in the table.

There were no significant age and ethnicity differences between women with and without an LLI (Table 2). Apart from the timing of the first antenatal visit all other characteristics compared between women with and without an LLI in the Table 2 were statistically significantly different.

**Table 2:** Socio-demographic and pregnancy characteristics of women with and without a limiting longstanding illness

**Characteristics**	**Limiting Longstanding Illness**		**P value**
	Yes (n = 1,705*)	No (n = 16,527*)	
	% [95%CI]	% [95%CI]	
**Age at delivery (years)**			p = 0.55
<25	22.3 [20.0,24.7]	21.5 [20.1,22.9]	
25-34	56.2 [53.7,58.8]	57.8 [56.7,58.8]	
35+	21.5 [19.2,23.9]	20. 8 [19.5,22.2]	
**Ethnic group**			p = 0.90
White	88.4 [85.2,90.9]	88.5 [86.1,90.6]	
Other ethnic group	11.6 [9.1,14.8]	11.5 [9.4,13.9]	
**Timing of the first antenatal visit**			p = 0.70
≤12 weeks	74.1 [71.3,76.6]	75.5 [74.2,76.9]	
13-18 weeks	18.3 [16.0,20.9]	17.0 [16.0,18.0]	
19+ weeks	4.4 [3.4,5.8]	4.4 [4.0,4.8]	
Did not receive antenatal care	3.2 [2.3,4.3]	3.1 [2.5,3.8]	
**Lone Parent**	19.5 [17.2,22.0]	13.9 [13.0,15.0]	p < 0.001
**Partner has used force in the relationship**	6.0 [4.5,7.8]	3.3 [2.9,3.6]	p < 0.001
**Family income below 60% of the national median (income poor)**	41.4 [38.1,44.7]	28.1 [26.3,30.0]	p < 0.001
**Self-rated family financial status**			p < 0.001
No financial difficulties	47.4 [44.3,50.6]	64.5 [63.1,65.8]	
Just about getting by	33.8 [31.2,36.4]	26.0 [25.0,27.1]	
In financial difficulties	18.8 [16.7,21.2]	9.5 [8.9,10.1]	
**Receiving means-tested benefits**	49.3 [46.1,52.6]	35.3[33.3,37.3]	p < 0.001
**Not owning home**	50.0 [46.4,53.6]	36.8 [34.9,38.7]	p < 0.001
**Highest educational qualification**			p < 0.001
Higher degree/ First degree/diploma	20.6 [18.1,23.4]	27.8 [25.6,30.1]	
A/AS/S levels	8.8 [7.4,10.4]	9.8 [9.2,10.4]	
O level/GCSE grades A-C	35.5 [32.7,38.4]	34.6 [32.9,36.3]	
GCSE grades D-G	12.8 [11.0,14.9]	10.7 [9.9,11.5]	
Other academic qualifications (including overseas)	2.0 [1.3,3.0]	2.3 [2.0,2.8]	
None	20.4 [18.2,22.7]	15.0 [13.7,16.3]	
**General health**			p < 0.001
Excellent/good	41.4 [38.5,44.3]	87.8 [87.1,88.5]	
Fair/poor	58.6 [55.7,61.5]	12.2 [11.5,12.9]	
**Pregnancy was a surprise**	50.5 [47.6,53.4]	42.0 [40.7,43.3]	p < 0.001
**Kept smoking during pregnancy**	29.2 [26.5,32.1]	20.8 [19.5,22.1]	p < 0.001
**This was the first baby**	36.6 [34.0,39.3]	42.4 [41.3,43.5]	p < 0.001
**Mode of delivery**			p < 0.001
Vaginal/instrumental	73.5 [71.1,75.7]	79.1 [78.3,79.9]	
Caesarean	26.6 [24.3,29.0]	20.9 [20.1,21.7]	
**Gestational age**			p < 0.001
Very preterm <32 weeks	1.6 [1.0,2.6]	0.9 [0.8,1.1]	
Preterm 32-36 weeks	9.3 [7.7,11.1]	5.9 [5.4,6.3]	
37-42 weeks	89.1 [87.0,90.9]	93.2 [92.8,93.7]	
**Duration of breast feeding**			p < 0.001
Never breast fed	33.5 [31.0,36.0]	30.1 [28.3,31.9]	
< 1 week	12.2 [10.5,14.2]	10.5 [9.6,11.4]	
1 week - 3 months	28.7 [26.3,31.3]	26.1 [25.1,27.0]	
> 3 months	25.6 [22.9,28.4]	33.4 [31.4,35.5]	
**Child has a limiting longstanding illness at 7 years of age**	12.0 [10.0,14.3]	6.2 [5.7,6.8]	p < 0.001

* Numbers weighted for the survey design and non-response.

Nearly one fifth of women with an LLI were lone parents compared to 13.9% of women without an LLI. Six percent of disabled women who had a partner said that the partner had used force in their relationship, such as grabbing, pushing, shaking, hitting or kicking compared to 3.3% of women without an LLI. The difference between the two groups could be even greater as 4.9% of disabled women did not want to say whether the force had been used compared to only 2.2% of women without an LLI.

A significantly higher proportion of women with an LLI lived in poverty, with almost half of families where the woman was disabled being on means-tested benefits compared to just over a third of families where the woman did not have an LLI. There were also inequalities in terms of the highest educational qualification achieved with a higher proportion of women without an LLI (27.8%) having a university degree or diploma compared to women with an LLI (20.6%).

A total of 58.6% of women with an LLI rated their general health as fair or poor compared with 12.2% of women without an LLI. About half of the pregnancies in women with an LLI were a surprise, while women without an LLI were slightly but significantly more likely to have a planned pregnancy. A higher proportion of disabled women continued smoking throughout pregnancy compared to women without an LLI.

Regarding pregnancy outcomes, disabled women were more likely to have a caesarean section and a preterm birth than women without an LLI. Babies born to mothers with an LLI were also less likely to be breast fed for more than three months compared to babies of mothers without an LLI.

With respect to the child health at the age of seven years (as reported by the main respondent, usually child’s mother), twice as many children of mothers with an LLI had an LLI themselves, compared to children of mothers without an LLI. The most common LLIs in children were diseases of the respiratory system (particularly asthma). Of all children with an LLI, 35.2% were reported to have a respiratory disease. Mental health and behavioural disorders (such as pervasive developmental disorders) and diseases of ear and mastoid process (such as hearing loss) were also relatively common (13.5 and 8.1% of all LLIs, respectively).

## Discussion

We found a prevalence of LLI of 9.4% in this population with musculoskeletal, respiratory and mental disorders accounting for most of the health problems. We were unable to identify any UK population data estimating levels of disability in pregnancy and indeed the MCS data reported here were collected nine months after the woman had given birth.

The LLI prevalence found in this study is similar to the LLI prevalence in women of childbearing age in general population found in other surveys in the UK. The prevalence of disability estimated in different studies varies depending upon the study design, the instrument used to measure disability, threshold definitions and the characteristics of the population. The prevalence of LLI in the Population Census 2001 for England in 16-44 years old women was 8% [8]. The Health Survey for England in 2001 reported a somewhat higher LLI prevalence ranging between 13 and 19%. When more specific disability questions were asked, 5-9% of 16-44 year old women were reported to have a moderate to severe disability [9]. The overall LLI prevalence reported in 2009 by the Health Survey for England was lower than in 2001 (13%, 9% and 15% in 16-24, 25-34 and 35-44 year old women, respectively) [10], but nevertheless in a range comparable with our findings as majority of women giving birth in the MCS were 25-34 years old.

Diseases of respiratory and musculoskeletal system and mental disorders are most frequently self-reported longstanding illnesses in women aged 16-34 years in the general population, with musculoskeletal problems becoming the most prevalent longstanding condition in women 35-44 years of age [10]. This is in line with our findings with back disorders, asthma and recurrent depressive disorder being the most common LLIs in women who recently had a baby.

A strength of this study is that the findings are based on a very large nationally representative dataset; weights were employed to adjust for study design and non-response. The characteristics of disability in women who had recently given birth were identified using an approach utilised in previous population surveys. However, it remains possible that some degree of underreporting of LLI may have occurred, for example for stigmatising LLIs such as disorders associated with drug use, schizophrenia, and intellectual disabilities. The main results, nevertheless, are consistent with findings in women of childbearing age. The study also relied on self-reporting of activity limiting longstanding health conditions with responses to some extent dependant on individuals’ understanding and interpretation of the question and perceptions of their own health. Even though respondents were asked to report only longstanding illnesses and it is reasonable to assume that most of them were present also during pregnancy, due to the timing of the interviews some LLIs, however, may have arisen after the birth of the child.

Over half of pregnancies in disabled women were not planned. This has implications for reproductive healthcare, including helping to choose the most suitable contraceptive method to meet the individual needs of women with disabilities.

Being disabled was associated with an increased risk of living in poverty. It is unclear from these data what is cause and what is effect. Other research suggests that disability is linked to poverty bidirectionally [3]. Living in poverty and associated disadvantage may lead to developing health conditions that can result in disability [11]. Acquisition of disability may worsen person’s economic and social wellbeing through a number of routes [12,13] including increased costs due to disability (e.g. for personal support and assistive devices), barriers to further education and employment, and reduced income. Disabled women were more likely to have no educational qualifications, be lone parents and suffer from general ill health. Poverty is a major determinant of ill health and disability; further efforts to reduce the social gradient and establishing a minimum income for healthy living are required [14].

A higher proportion of children of mothers with an LLI were born preterm and were reported as having an LLI at the age of seven years, which could be due to a number of individual factors and wider determinants of health including increased likelihood of disadvantage experienced during pregnancy and the early years. From a clinical point of view, two findings are of particular importance: women with an LLI were more likely to keep smoking throughout pregnancy and they were also more likely to experience intimate partner violence. Smoking in pregnancy is a known risk factor for a number of pregnancy and birth related problems (including complications during labour, stillbirth and premature birth) and child health problems (including low birth weight, sudden infant death syndrome, respiratory infections and asthma, ear infections and behavioural problems) [15]. Smoking cessation services should be sensitive to the often disadvantaged circumstances of pregnant disabled women and aim to provide ongoing support working in partnership with other agencies providing support for these women. Intimate partner violence during pregnancy can lead to perinatal death, preterm birth and low birth weight [16,17]. Women experiencing intimate partner violence can find it difficult to disclose the abuse to the healthcare professionals and attend antenatal appointments. Healthcare professionals should undergo training to recognise features of intimate partner violence and the antenatal services should be commissioned which allow flexibility in antenatal appointments [18].

Babies of mothers with an LLI were also less likely to be breast fed for more than three months adding further to disadvantage in this group. For example, infants who are not breastfed are more likely to acquire respiratory infections and gastroenteritis. It is recommended that an ongoing support paying attention to the specific needs of women who are less likely to breastfeed is provided [19], including for women with LLIs.

## Conclusions

Nearly ten percent of women who have recently given birth in the UK report some degree of disability as measured by the presence of an LLI. Disability associated with chronic health conditions, such as back problems, asthma and recurrent depressive disorder is relatively common.

There are significant associations between maternal and child LLI, as well as health behaviours and wider health determinants including socio-economic factors. Women with disability should have access to comprehensive family planning services that take into account their individual circumstances. Apart from condition-specific support from health professionals during and after pregnancy, women with an LLI may require extra help to quit smoking and to continue breast feeding. These findings also underline the need to identify and provide support for all women experiencing intimate partner violence of which women with an LLI are at greater risk. Children of women with an LLI were more likely to be born in poverty which is an important determinant of ill health and further efforts to reduce income inequality and establishing a minimum income for healthy living are required. We plan further analytical work to explore the complex inter-relationships between maternal LLI, poverty and child health outcomes.

While this brief report provides a general overview on disabilities in pregnancy and associated factors, a comprehensive study to measure different dimensions of disability is recommended in order to identify groups of women with specific needs during pregnancy and to help clinicians and maternity service providers to tailor their services to meet these needs. In this paper, we restricted analysis to data on LLI which are available in the MCS. A more rounded analysis of disability, however, would consider social and environmental responses to an LLI and the impact which this has on an individual’s capacity to participate fully in society; such disability research in relation to maternity care would need to consider the way healthcare providers including obstetricians and midwives respond to women with disability.

## Competing interests

Authors declare that they have no competing interests.

## Authors’ contributions

All authors (DŠ, JJK, MER, RG) contributed to the design of the study and interpretation of data. DŠ performed the data analysis and drafted the manuscript. All authors (DŠ, JJK, MER, RG) critically revised the manuscript, and read and approved the final version.

## Pre-publication history

The pre-publication history for this paper can be accessed here:

http://www.biomedcentral.com/1471-2393/12/31/prepub
